# First-year college students’ weight change is influenced by their randomly assigned roommates’ BMI

**DOI:** 10.1371/journal.pone.0242681

**Published:** 2020-11-24

**Authors:** Irene van Woerden, Daniel Hruschka, Alexandra Brewis, David R. Schaefer, Meg Bruening

**Affiliations:** 1 Department of Community and Public Health, Idaho State University, Pocatello, ID, United States of America; 2 School of Human Evolution and Social Change, Arizona State University, Tempe, AZ, United States of America; 3 Department of Sociology, University of California–Irvine, Irvine, CA, United States of America; 4 College of Health Solutions, Arizona State University, Phoenix, AZ, United States of America; Pennington Biomedical Research Center, UNITED STATES

## Abstract

**Background:**

There is ongoing debate about whether friends’ greater similarity in Body Mass Index (BMI) than non-friends is due to friend selection, shared environments, or peer influence.

**Methods:**

First-year college students (n = 104) from a southwestern U.S. university were randomly assigned roommates during the university’s housing process, effectively removing friend selection effects. Participant BMI was measured up to four times (T1-T4) across 2015–2016. The influence of roommate baseline BMI (T1) on change in participant BMI over time (T2-T4) was analyzed using a linear mixed effects model adjusted for individual socio-demographics, linear time trends, baseline BMI, and physical clustering of students. A sensitivity analysis examining floormates was also conducted.

**Results:**

Consistent with roommate influence, participants randomized to roommates with a higher BMI gained more weight between times T2 and T4 (β = 0.06; 95% CI = 0.02, 0.10). No shared environment effects (shared campus or floor) were found.

**Conclusions:**

Randomly assigned roommates influenced each other's weight trajectories. This clarifies that BMI convergence can occur outside of friend selection or shared environments mechanisms.

## Introduction

Consistent evidence suggests that Body Mass Index (BMI) is similar among friends [1–3]. There are three proposed mechanisms for how this greater similarity among friends arises. First, BMI may affect who one chooses to befriend [3–6]. Second, once friendships have developed, shared beliefs and activities might create convergences as behaviors increasingly mimic each other [3, 6]. Third, an indirect effect of shared environments or unobserved characteristics may result in similar BMIs [7, 8]. Determining how these three mechanisms contribute to observed similarities in BMI is challenging due to complex processes of friendship formation and change [7]. These mechanisms may co-occur and should thus be evaluated in light of one another.

The current study aims to determine if peer influence on BMI occurs among college roommates. In this study we examine how the BMI trajectory of first-year college students living on campus is associated with the baseline BMI of their roommate. College roommates are unique as they share a physical residence and inevitably spend some time together, but are not necessarily friends. While this current study does not propose specific mechanisms as to why peer influence on BMI occurs, it does present methods for ruling out two common confounders of social network studies—peer selection and environmental effects–and hence isolates influence as a mechanism.

College marks an important time and context to study peer influence on BMI for several reasons. Entering university is associated with weight increases [9] and US college populations are presenting with higher mean body weights than in the past [10]. First-year college students are at the start of the emerging adulthood period and typically have a much higher degree of autonomy than prior to starting college [11]. High school students are more likely to have an adult aware of their behaviors than college students [12]. While youth who have not yet started college may spend large amounts of time with peers, the ability to make decisions on weight-related behaviors (e.g., food choices, alcohol use, sleep patterns) while still living in their natal household may be limited [13–16]. It is suggested that high school students’ eat around four meals with their family each week [17]. In comparison, college students living away from home are limited in how many family meals they can share [18]. This difference in shared family meals suggests that, with the likely exception of lunch, adolescents will be less a part of their friends’ meals than are college students who live together. Moreover, as students transition from high school to college they tend to make less healthful choices, such as engaging in less physical activity, consuming less fruits and vegetables, and consuming more alcohol than when in high school [19, 20]. College students typically have less structured classroom time than adolescents in high school [21], resulting in greater flexibility in choosing what they do, and when, where, and how often they do it, than adolescents in high school. This provides the opportunity for learning not just behaviors, but how others organize their life and the priority various behaviors are given. Thus, we expect increasing opportunities for social learning as individuals move away from home for the first time.

While prior studies have identified a role for peer influence in many health and well-being outcomes [3, 6, 22–28], it can be difficult to disentangle peer influence from peer selection and environmental effects. We take two approaches to rule out these competing explanations. First, if college roommates are more similar than non-roommates, similarities in BMI trajectory may be due to pre-existing similarities that affected roommate choice, rather than peer influence. We overcome this confound by studying a sample of roommates who were randomly assigned. This strategy effectively rules out peer selection as the mechanism for any BMI similarities. Second is another main confounder of peer influence: shared environment. Roommates may be similar to each other due to spending time in the same environment. To test if roommate influence was due to a shared environment, we repeat our analysis, but with participants randomly paired to other participants who reside on the same (or nearby) floor. If we find evidence of similarity among randomly assigned floormates, then this suggests shared environment is responsible. But, if peer influence is not found when examining these pseudo-roommates then there is little evidence for a shared environment effect.

We study how roommates affect one another’s BMI trajectories using a sample of first-year college students living with randomly-assigned roommates in multi-story dormitories. Data were collected at four time points across an academic year, allowing us to test for change in BMI as our outcome. Unlike most prior studies that use self-reports of weight, we measure BMI. Self-reported weight tends to be under-reported at higher weights, and over-reported at lower weights [29, 30]. By using measured BMI these reporting errors are removed. The design of our study (i.e., random assignment) precludes selection as a source of similarity in BMI among roommate pairs, whereas our floormate analysis evaluates shared environment, allowing us to offer an innovative analysis of peer influence among college students. Moreover, by focusing on college roommates, and not high-school friends, we offer a test of peer influence occurring later in the life course and within a relatively underexplored type of relationship.

## Background

Numerous studies have shown that friends' BMI converges over time [3, 6, 22], and researchers have proposed several explanations for this convergence based on peer influence. One set of explanations focuses on *what* is spreading when influence occurs. Studies have suggested that convergence on BMI occurs indirectly because of peer influence on weight-related behaviors [31, 32]. For example, convergence of physical activity [3, 6], screen time [6, 25], disordered eating [23], food choices [24, 25], alcohol consumption [26], weight control behaviors [27], and sleep patterns [28] has been observed among friends. While these studies all showed peer influence on weight-related behaviors, only Simpkins tested if peer influence on weight-related behaviors was responsible for similarity in BMI among peers. Simpkins et al. examined if convergence of physical activity levels explained the convergence of BMI among adolescents, after controlling for selection effects [3]. While Simpkins reported peer influence for both BMI and physical activity, there was no evidence the similarity in BMI was due to the similarity in physical activity [3]. Another study examining peer influence among college students suggested that while friends tend to have similar meal consumption patterns, fruit and vegetable intake, alcohol intake, sleep behaviors, and stress levels, only dining hall use and stress levels were also associated with BMI change; hence, only dining hall use and stress levels had the *potential* to explain why friends BMI tends to be similar [33]. Alternatively, it may be the case that norms, not behaviors, spread through social networks and these lead to convergence in weight [1, 34]. For instance, friends may influence the perception of what a 'socially acceptable' weight is, indirectly resulting in weight change [32].

The second set of social influence theories focus on the question of *how* influence on weight occurs. This is important because the processes related to influence may play out differently depending upon the type of relationship in question or life cycle stage. Much research on peer influence has focused on friendship or other affective relationships and proposed that processes such as social learning [35] are responsible. For instance, prior studies suggest that learning from media figures may result in children spending more time exercising [36], learning from coworkers may be associated with smoking behaviors and BMI [37], and family and friend support may be associated with physical activity levels [38].

Peer influence via social learning requires that individuals observe others’ behaviors that are salient to the outcome. In regards to weight-related behaviors, the capacity to observe others can differ markedly for friends vs. roommates. Namely, roommates may see the kinds of food each other brings to the room and observe workout, eating, and sleeping schedules. Additionally, given the shared living space, roommates may observe each other during periods when withdrawal from friendship networks is expected to occur, such as periods of depression [39]. For students who tend to avoid social situations, such as students with social anxiety [40], the extent of roommate observation may be higher than other peer observation. If a friendship develops then roommates may also shop and eat together. While friends may observe and participate in some of these activities, friends who do not live together do not have the capacity to directly monitor one another’s behavior at the same intensity as do roommates. Even if a friendship *per se* does not develop among roommates, they still have greater access to one another’s “backstage” selves, compared to the “frontstage” selves that individuals present to their friends and acquaintances [41].

Peer influence via social learning also requires that individuals remember, and later reproduce, others’ behaviors [42]. Compared to friends, roommates may be optimally placed to learn behaviors from each other. Roommates may spend much of their time together while engaged in routine activities (e.g., morning and evening routines) which can be highly repetitive. The repetitiveness of roommates interactions may result in behaviors which would typically go unnoticed being remembered, and reproduced. In comparison, if friends engage in a wider variety of shared activities it may be harder to notice, remember, and reproduce each other’s behaviors.

In contrast to social influence explanations, friends may have similar weight because people tend to select friends who are already similar to themselves (i.e., homophilous selection) [3–6]. Friendships may form on the basis of body size, with homophily arising directly via selection into friendships based on similar weight status. Homophilous selection may also occur at a genetic level, with people who have similar biological characteristics more likely to become friends [43, 44]. Given that some people may be more susceptible to obesity than others due to genetics [45, 46], such selection could result in similarity in BMI among friends.

Friends may also have similar BMIs due to *not* being selected into friendships by others with a higher or lower BMI. Systematic studies show discrimination against individuals with very high BMI [47]. The stigma of obesity may decrease friendship opportunities for individuals who are overweight/obese [4, 5]. As a consequence, individuals categorized as overweight or obese may turn to one another for friendship, resulting in similarities in BMI among friendship groups. Similarly, friendship discrimination may occur among those who are underweight [22].

The third explanation for weight similarities among friends is shared environment. Geographic location has been associated with friendships, with those living closer together more likely to be friends [48]. Similarities in BMI have been observed with people living in the same zip code [49] and county [50]. Thus, both friendships and BMI are spatially clustered. As a consequence, friends’ BMI may converge because living in close geographic proximity provides common opportunities for physical activity and food consumption. Aspects of the environment that might lead to converging BMI include built environments (e.g. parks, walkable neighborhoods) that encourage or discourage physical activity [51–53] and the availability of different kinds of food outlets [54, 55]. Thus, when friends reside in the same environment, their BMI may converge over time due to exposure to a common environment rather than any direct influence between friends. This possibility is particularly salient for roommates who share a residence during the academic year.

Given the aforementioned issues, concern has been raised about whether it is influence via friendship ties or other mechanisms that explain the BMI convergence observed in social network studies [7, 8]. Causal inference regarding peer influence is complicated by the fact that homophily based on BMI among friends can arise through multiple mechanisms. To overcome this concern, one approach has been to statistically control for selection into friendships when examining BMI change such as by using stochastic actor-oriented models (SAOMs) to simultaneously examine selection and influence. Following this approach, researchers have found influence on BMI among high-school students [3, 6] and university students (Bruening et al. 2018) while controlling for selection into friendships based on similarity in BMI. Together, these studies offer compelling evidence for peer influence, but nonetheless, make the strong assumption that all confounding environmental factors are specified in the model.

A second approach to overcoming concerns about selection has been to use an experimental design. By randomly assigning individuals to a relationship, selection effects are effectively removed. This is important given that peer selection is a major confounder in observational studies of peer influence—it is difficult to fully control for all of the variables (some unobservable) which may result in peers choosing to be friends or not. When participants are randomly assigned to each other, relevant confounders and omitted variables that might exert influence on friend selection are eliminated and the only mechanisms that can systematically create similarities among them are peer influence and environmental effects. It is not often that researchers can randomly assign people to long-term relationships. However, the random assignment of first-year student roommates practiced by some universities offers a suitable natural experiment. Research using randomized first-year college student roommates has shown roommates influence each other’s verbal SAT scores [56], and grade point average [57]. To our knowledge, only two studies have examined the association of randomly assigned roommate BMI and participant BMI change. The first study, conducted among female students at a private Midwestern university, found no effects of roommate BMI on participant BMI trajectories, and a *negative* association between a participant's weight change and their roommate's weight (i.e. they concluded that first-year students randomly assigned to a heavier roommate, gained *less* weight over the academic year) [58]. The second study included males and females from a large private and a large public university; this revealed a *positive* association between participant’s weight change and their roommate’s weight for females, but not males [59]. It is unclear why these studies produced mixed findings. One reason may be due to the calculation of BMI using self-reported weight and height which can lead to measurement error [29] and make it more difficult to detect the effects of peer influence. Additionally, some of the students in one of the studies had more than one roommate, which may complicate roommate influence [59].

Given findings contrary to predictions derived from the broader literature on friendship and BMI change, a more detailed study is warranted. We replicate the quasi-experimental approach in prior studies, but with several improvements. We recruit a more diverse student population, have better follow-up over the course of the year (i.e., up to four observations per participant), only include participants with roommates in the study, and only include roommates in double occupancy rooms (there were no triple- or quad- occupancy rooms available). We also measure anthropometrics directly, rather than relying on self-reports of height and weight to calculate BMI. Given a prior roommate study examining weight change found peer influence for females but not males [59], we include both males and females. Sex differences in several weight-related factors exist. For instance, normal weight women are more likely to believe they are overweight than normal weight men [60], and women are more likely to engage in weight loss than men [60, 61]. There are indications that peer influence may be sex-specific [59, 62–65]. Lastly, we develop an analytic method to rule out shared environment as a competing explanation, resulting in an even stronger study design than used previously.

## Methods

### Study sample

Study data were collected as a core component of the longitudinal Social impact of Physical Activity and Nutrition in College (SPARC) study of 1435 first-year students from Arizona State University [66]. At Arizona State University all first-year freshmen are expected to live on campus, and housing is guaranteed for all first-time freshmen [67]. The university has four campus locations, each with at least one residence hall for students. The number of floors in each residence hall varied from three to fifteen. Residence halls are limited to students of specific colleges; however students of each college typically have multiple residence hall options [68]. Students can request a roommate of the same-sex whose major is also housed in the academic college. If students do not request a roommate they complete a short survey (including questions such as cleanliness and bedtime) and are randomly assigned to a roommate (in theory to someone with a similar survey response). Students are not asked about their weight or demographics in the short survey, and hence roommates are not assigned based on their weight or demographics.

Data collection took place at the start and end of the fall 2015 and spring 2016 semesters (August 2015, November 2015, January 2016, April 2016), with the same items measured at each observation. Participant recruitment occurred primarily at the start of the semester during the participants’ weekly floor meetings; recruitment was not targeted to roommates. Participants were recruited from all four university campuses, however the majority of students were recruited from two of the campuses (labeled A and B). Some students not living in a residence hall also participated in the main study. Participant follow up occurred in-person and via web-based communications. Inclusion criteria for the main analyses were: (1) baseline completion of survey and anthropometrics (start of fall 2015) and at least one re-sampling in the spring 2016 semester; (2) roommate completed the same baseline measures; (3) baseline BMI value within three Standard Deviation (SD) of the mean sample BMI (so that students with the highest BMI values would not overly influence the results; two roommate pairs were excluded due to this criteria); (4) roommate BMI within three SD of the mean BMI; (5) first year student residing on campus A or B (only one roommate pair was excluded due to this criteria); (6) no history of friendship or other substantive acquaintance between roommates prior to campus co-residence; (7) roommate stability throughout the academic year. The total sample size decreased over time with 104, 93, 96, and 80 participants at times 1 to 4, respectively. Informed consent was obtained and all study protocols were approved by the Arizona State University Institutional Review Board.

### Measures

#### Body mass index (BMI)

Height and weight measurements were obtained by trained research staff using portable SECA scales and stadiometers to the nearest 0.1kg and 0.1cm, respectively. BMI was calculated as kg/m^2^; participant weight status was classified as clinically-defined overweight/obese if their BMI was ≥ 25 kg/m^2^ [69].

#### Roommate measures

Mutual reports of campus, residence hall, floor, and room were used to identify roommates and co-living location. This was confirmed by triangulation with university records. To identify and remove roommates with any history of friendship or other substantive acquaintance prior to campus co-residence the relationship between roommates was obtained. Each participant listed five male and five female friends at the university, and how they knew each other (boyfriend/girlfriend, family member, friend before college, met for the first time at the university, or other). Participants who stated their roommate was a boyfriend/girlfriend, family member, friend before college, or known some other way prior to the start of college, were excluded from the analyses. To determine if participants stayed roommates throughout the academic year, respondents at times two to four were asked "Have you moved since the last time you took this survey?", and "Has your roommate moved since the last time you took this survey?" If a participant answered affirmatively to either question, both the participant and their roommate were excluded from the analyses.

#### Shared environment and floor-level assignment

To assess whether influence among roommates could have been due to shared environment, we conducted an analysis that matched individuals with non-roommate floormates. If shared environment accounted for the increased convergence among friends, then convergence among matched floormates should be observed. This analysis of non-roommate floormates also provides a check on whether the assignment of majors to the same floor may account for convergence among roommates. Specifically, if greater BMI convergence among roommates is due to assignment of majors to the same floors, then we should also observe convergence among matched floormates. All participants who met the inclusion criteria for the main analyses were randomly matched to a student who (1) had not met the inclusion criteria for the main analyses (2) was the same sex as the participant (3) was living in the same residence hall as the participant (4) had a completed a baseline survey, a known baseline BMI, and at least one known BMI in the spring 2016 semester and (5) had a baseline BMI within three SD of the mean. Participants were matched to someone who lived on the same residence floor when possible. In the cases (n = 33) when there were more students in the main analysis than students on the same residence floor who met the inclusion criteria, students in the main analysis were matched to a student from a nearby residence floor (e.g., the floor above/below the students residence floor) who met the inclusion criteria.

#### Socio-demographic characteristics

Participants’ sex, financial aid status, and race/ethnicity were obtained via self-report. Race/ethnicity was dichotomized as Non-Hispanic White vs. not. Financial aid status was based on whether or not the respondent had a Pell Grant (a federal grant for low-income students) or not, with the former a proxy for lower family socioeconomic status (SES).

### Statistical analysis

Chi-square and t-tests were used to compare the demographics of the participants included and excluded from the roommate analysis. A comparison of roommate and floormate demographics was conducted to assess the randomization of roommate demographics (self-identified race/ethnicity, Pell Grant (financial aid) status, measured BMI, and classified weight status based on BMI). The analysis considered how often roommates and floormates shared attributes (for race/ethnicity, Pell Grant status, weight status), and their mean absolute difference (for BMI).

To assess the influence of roommate BMI on one’s own BMI over the course of the academic year, we fit a linear mixed effects model. A linear time effect included estimation of participants’ BMI trend over time (model A).

Model A: BMI_(participant)T2,T3,T4_ ~ Sex + Race/ethnicity + Pell Grant status + campus + BMI_(participant)T1_ + BMI_(roommate)T1_ + BMI_(participant)T1_*Time + BMI_(roommate)T1_*Time + (1|person/room)

As participants’ BMI trajectory may be associated with their demographics [70, 71], controls for sex, race/ethnicity, and Pell Grant status were included. To examine if campus location predicted a different BMI intercept, an adjustment for campus was included. Participant and roommate baseline BMI, and a linear time interaction with participant and roommate baseline BMI, examined how participant and roommate baseline BMI were associated with the participants' BMI intercept and trajectory. We included participant baseline BMI in the model to control for any association between initial BMI and BMI change. To account for the repeated observations per person and within each room, repeated measurements of BMI were treated as clustered within persons within rooms. Both participant and roommate baseline BMI were centered at a BMI score of 25 to facilitate interpretation of model effects.

Several sensitivity analyses were conducted. To examine the potential effect of participant sex and environment on their BMI trajectory, a sex by time interaction term, and a campus by time interaction term, were included (model B).

Model B: BMI_(participant)T2,T3,T4_ ~ Sex + Race/ethnicity + Pell Grant status + campus + Sex*Time + campus*Time + BMI_(participant)T1_ + BMI_(roommate)T1_ + BMI_(participant)T1_*Time + BMI_(roommate)T1_*Time + (1|person/room)

To further examine shared environment effects a model controlling for residence hall rather than campus was run (model C). To examine if the results were consistent when using another measurement of roommate body size, we estimated a model including roommate weight status (overweight/obese or not) instead of roommate BMI as a predictor (model D). To ensure that the results were consistent when participants’ baseline BMI was excluded as a predictor, an additional model predicting participants T1-T4 BMI by participant demographics and roommate BMI was run (model E). To better examine energetic changes, model A was also run using body weight, rather than BMI (model F). While the sample size was small, model A was also run stratified by sex, rather than controlling for sex, to examine potential differences by sex in detail (model G). We also stratified analyses by semester to determine if the effect was isolated in a specific semester. We did this by predicting BMI at the end of the Fall semester by participant and roommate BMI at the start of the Fall semester (model H), and predicting BMI at the end of the Spring semester by participant and roommate BMI at the start of the Spring semester (model I). Finally, to distinguish potential peer influence from environmental effects, the model was rerun using the randomly matched floormates, rather than the participants’ roommate (model J).

Model C: BMI_(participant)T2,T3,T4_ ~ Sex + Race/ethnicity + Pell Grant status + Residence Hall + BMI_(participant)T1_ + BMI_(roommate)T1_ + BMI_(participant)T1_*Time + BMI_(roommate)T1_*Time + (1|person/room)

Model D: BMI_(participant)T2,T3,T4_ ~ Sex + Race/ethnicity + Pell Grant status + campus + Weight status_(participant)T1_ + Weight status _(roommate)T1_ + BMI _(participant)T1_*Time + Weight status _(roommate)T1_*Time + (1|person/room)

Model E: BMI_(participant)T1,T2,T3,T4_ ~ Sex + Race/ethnicity + Pell Grant status + campus + BMI_(roommate)T1_ + BMI_(roommate)T1_*Time + (1|person/room)

Model F: Weight_(participant)T2,T3,T4_ ~ Sex + Race/ethnicity + Pell Grant status + campus + weight_(participant)T1_ + weight_(roommate)T1_ + weight_(participant)T1_*Time + weight _(roommate)T1_*Time + (1|person/room)

Model G: BMI_(participant)T2,T3,T4_ ~ Race/ethnicity + Pell Grant status + campus + BMI_(participant)T1_ + BMI_(roommate)T1_ + BMI_(participant)T1_*Time + BMI_(roommate)T1_*Time + (1|person/room). (Stratified by sex)

Model H: BMI_(participant)T2_ ~ Sex + Race/ethnicity + Pell Grant status + campus + BMI_(participant)T1_ + BMI_(roommate)T1_

Model I: BMI_(participant) T4_ ~ Sex + Race/ethnicity + Pell Grant status + campus + BMI_(participant)T3_ + BMI_(roommate)T3_

Model J: BMI_(participant)T2,T3,T4_ ~ Sex + Race/ethnicity + Pell Grant status + campus + BMI_(participant)T1_ + BMI_(floormate)T1_ + BMI_(participant)T1_*Time + BMI_(floormate)T1_*Time + (1|person/room)

A total of 208 participants were included in the floormate analyses at time 1. The number of participants included in the floormate analysis at times 2 to 4 varied each iteration, with a mean of 181, 189, and 158 participants, respectively. As the results could potentially change depending on the randomization, 100 different randomizations were run. The distribution of the model parameters of interest was used to determine the beta point estimate, confidence interval, and level of significance. The statistical software R (version 4.0.0) was used for all analyses. The statistical package "nlme" was used to estimate the models [72]. Statistical significance was determined at p<0.05.

## Results

**Fig 1:**
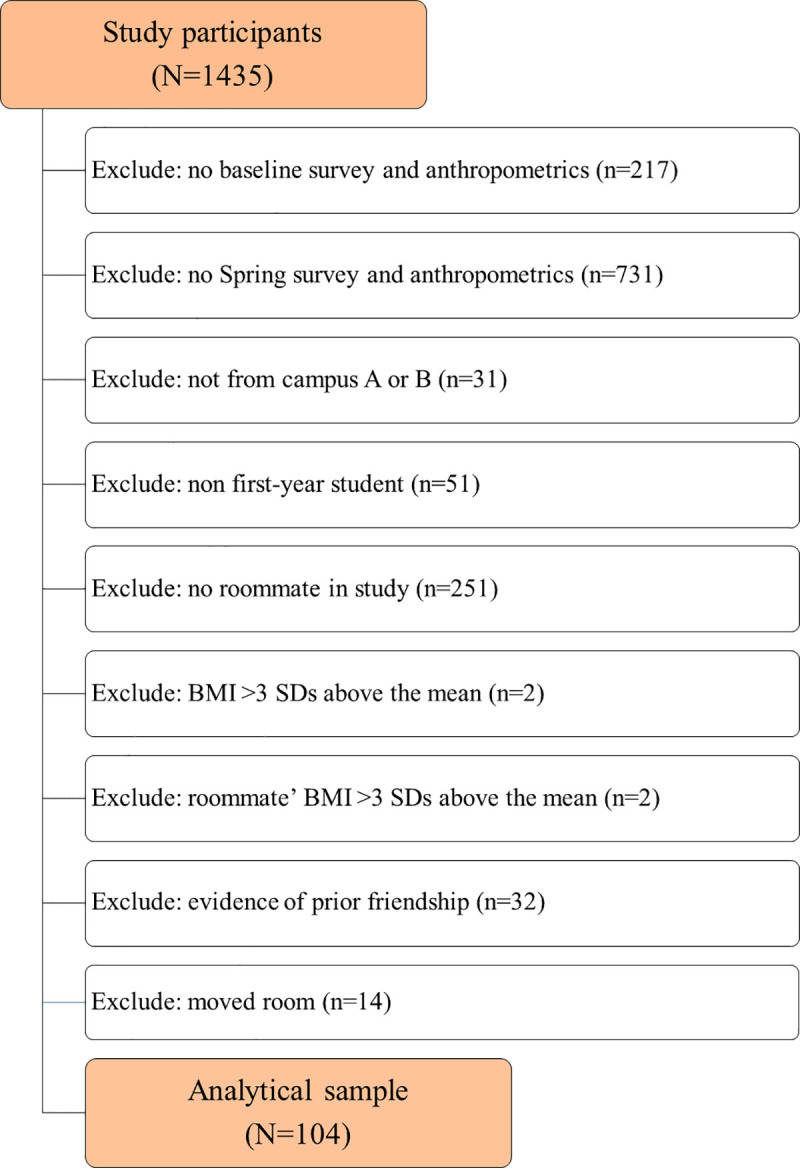
Flow chart of total study population and final analytical sample.

**Fig 2:**
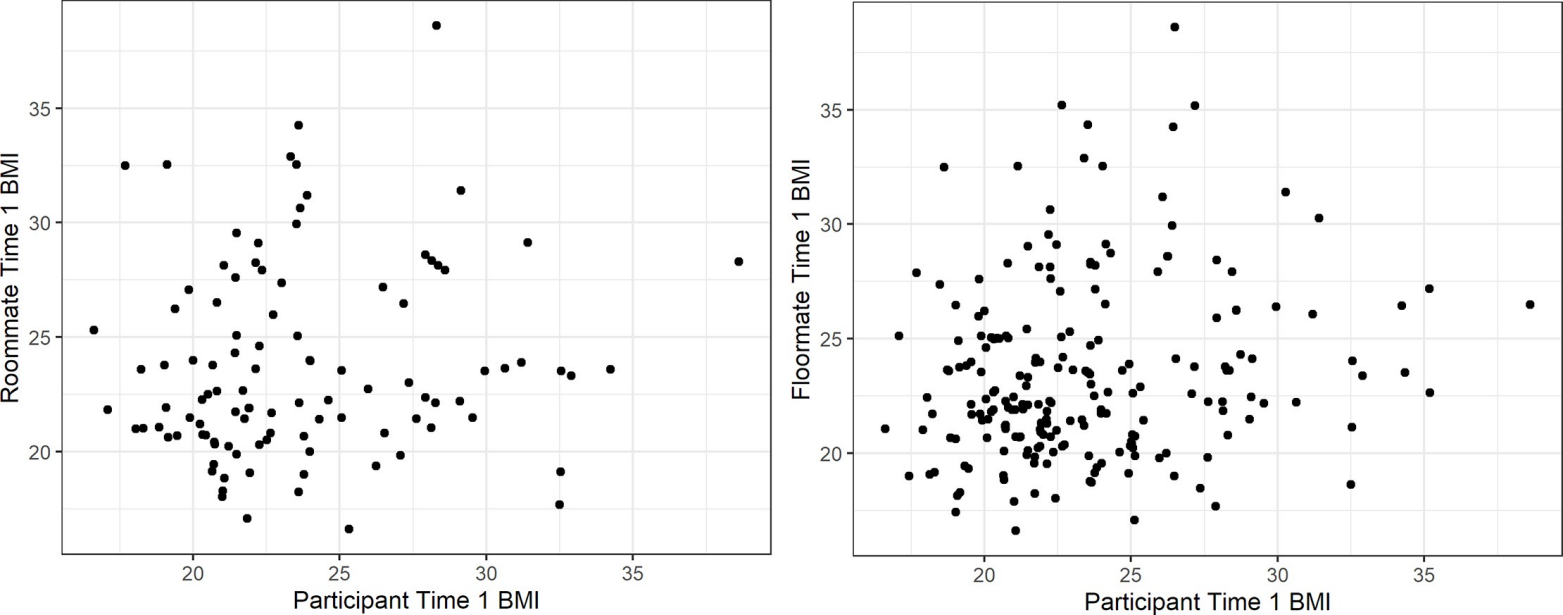
Correlation of participant and roommate, and participant and floormate, BMI at Time 1.

A total of 150 participants residing in 75 shared rooms met inclusion criteria 1 to 5 (Fig 1). There were 32 participants (16 shared rooms) excluded from the analysis as they did not meet the sixth inclusion criteria (no evidence of prior friendship), indicating that their roommate assignment may not have been random. An additional 14 participants (7 shared rooms) were removed from the analyses as these participants did not remain roommates throughout the academic year, potentially limiting influence effects (inclusion criteria seven). There was no evidence that the remaining 104 participants (81% female, 45% non-Hispanic White; Table 1) residing in 52 shared rooms, were friends prior to meeting each other on campus or changed roommates during the year. The majority (73%; 76/104) of the 104 participants reported their roommate as a friend, and stated that they met their roommate on campus. The remaining 27% (28/104) of participants did not nominate their roommate as a friend; how these roommates met is unknown. Compared to the students who were not included in the roommate analysis, the 104 students included in the roommate analysis were more likely to be female (P = 0.001) and from campus A (P<0.001), which reflects the higher participation rates of females and students from campus A (Table 1). No difference by race/ethnicity (P = 0.570), Pell Grant status (0.064), or BMI (0.414) was observed between the students who were included, or excluded from the roommate analysis. Participants with missing socio-demographic data, and participants without a measured baseline and Spring BMI, were omitted from the analysis. No imputation procedures were completed. Monte Carlo permutation results showed that the roommates were no more similar than floormates by Pell Grant status (*P* = 0.576), weight status (*P* = 0.460), or baseline BMI (*P* = 0.060). However, roommates were more likely to both be non-Hispanic White than floormates were (*P* = 0.048). The correlation of participant and roommate baseline BMI was 0.11, while the correlation of participant and floormate baseline BMI ranged between -0.17 and 0.38 (Fig 2). There were 70 students in the roommate analysis with complete data, 25 students with missing data at one time point, and 9 students with missing data at two time points. Two students had an unknown BMI at T2, 5 students had an unknown BMI at T3, and 18 students had an unknown BMI at T4. An additional 6 students had unknown BMI for T2 and T4, and 3 students had an unknown BMI for T3 and T4. No differences in gender (P = 1.000), race/ethnicity (P = 0.634), Pell Grant status (P = 0.198), or BMI (P = 0.468) between the students in the roommate analysis with complete versus incomplete data were found.

**Table 1:** Key demographics of the first-year college students’ who were included, and excluded, from the roommate analysis.

Total, n	Excluded (n = 1248)	Included (n = 104)	*P*
**Sex, n (%)**			**0.001**
Female	793 (63.5)	84 (80.8)	
Male	455 (36.5)	20 (19.2)	
**Race/ethnicity, n (%)**			0.570
Non-Hispanic White	621 (49.8)	47 (45.2)	
Non-Hispanic Black	109 (8.7)	9 (8.7)	
Hispanic	319 (25.6)	26 (25.0)	
Mixed/Other	199 (15.9)	22 (21.2)	
**Pell Grant recipient, n (%)**	399 (32)	43 (41.3)	0.064
**BMI, mean (SD)**	24.1 (4.9)	23.7 (4.2)	0.414
**Weight (kg), mean (SD)**	69.1 (16.5)	65.9 (15.1)	0.061
**Campus, n(%)**			**<0.001**
A	521 (41.7)	76 (73.1)	
B	727 (58.3)	28 (26.9)	

The correlation for the first iteration (of the 100 different randomizations) of participant and floormate BMI is shown here.

**Fig 3:**
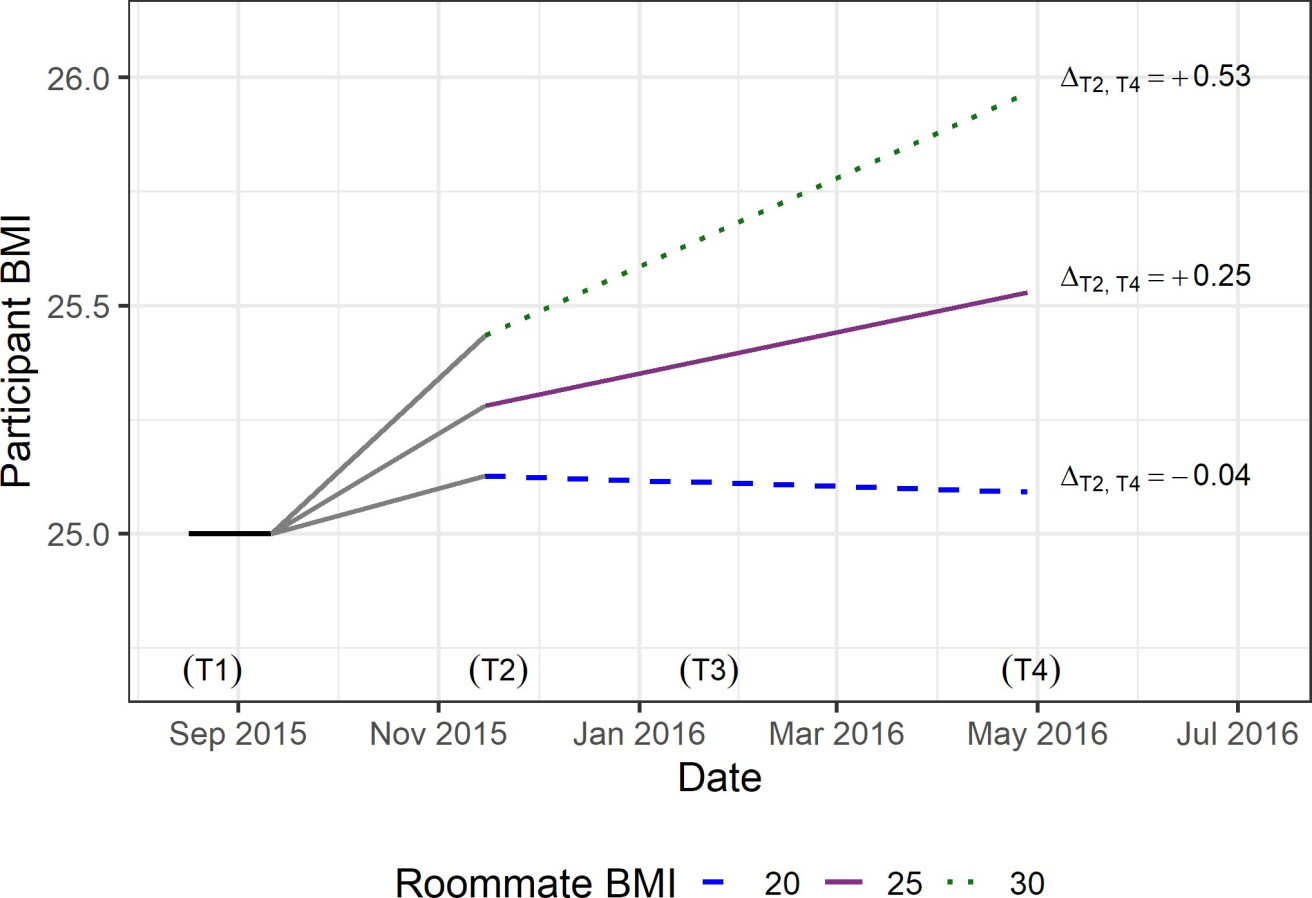
Predicted BMI trajectory over the academic year for a non-Hispanic White female participant whose baseline BMI was 25 by roommate BMI.

The linear mixed model showed student baseline BMI strongly predicted their BMI in later waves (β = 0.98, 95% CI = 0.93, 1.02; Table 2). Consistent with roommate influence, roommate baseline BMI significantly modified participant’s BMI trajectory over the school year (T2-T4), as indicated by a significant interaction between time and roommate BMI (β = 0.06; 95% CI = 0.02, 0.10). The model suggests that a participant with a baseline BMI of 25, and with a roommate with BMI of 20 would be expected to decrease 0.04 kg/m^2^ between T2 and T4, while a participant with a baseline BMI of 25 and with a roommate with BMI of 30 would be expected to increase 0.53 kg/m^2^ between T2 and T4 (Fig 3). These changes in BMI correspond to a 1.75m (5'7") participant with a T1 BMI of 25 losing 0.1 kg (0.2 lb), or gaining 1.6 kg (3.6 lb), between T2 and T4.

**Table 2:** The association of participant BMI change at a large southwestern university over the 2015–2016 academic year and roommate baseline BMI (n = 104).

		β	SE	95% CI	*P*
Intercept		25.57	0.18	(25.21, 25.93)	**<0.001**
Linear time trend^A^		0.25	0.09	(0.07, 0.43)	**0.008**
Sex	Female	(ref)			
	Male	-0.50	0.28	(-1.05, 0.05)	0.080
Race/ethnicity	Non-Hispanic White	(ref)			
	Other	-0.29	0.19	(-0.67, 0.09)	0.143
Pell Grant recipient	No	(ref)			
	Yes	0.06	0.19	(-0.32, 0.44)	0.753
Campus	A	(ref)			
	B	0.01	0.24	(-0.46, 0.48)	0.952
Participant BMI @ Time 1		0.98	0.02	(0.93, 1.02)	**<0.001**
Roommate BMI @ Time 1		0.03	0.02	(-0.02, 0.08)	0.209
Time^A^: Participant BMI @Time 1		0.02	0.02	(-0.02, 0.06)	0.328
Time^A^: Roommate BMI @Time 1		0.06	0.02	(0.02, 0.10)	**0.007**

^A^ The time variable in the model is from Time 2 (0, end of Fall semester) to Time 4 (1, end of Spring semester).

Boldface indicates statistical significance (p<0.05).

When time interactions for campus and sex were included in the model, the results did not change; time interactions for campus and sex were not significant (*P* = 0.224 and *P* = 0.401; S1 Table). Further, when residence hall, rather than campus, was included as a predictor in the models the results were consistent (S2 Table).

When roommate weight status was included in the model instead of roommate BMI, participant BMI trajectory remained significantly predicted by roommates’ weight status (β = 0.49, 95% CI = 0.12, 0.85; S3 Table). This suggests a participant with a baseline BMI of 25 with a non-overweight/obese roommate would be expected to increase 0.03 kg/m^2^ between T2 and T4. A participant with a baseline BMI of 25 and with an overweight/obese roommate would be expected to increase 0.51 kg/m^2^ between T2 and T4.

When participant T1 to T4 BMI was predicted by their roommate BMI, participant BMI was significantly associated with roommate baseline BMI (β = -0.98, 95% CI = -1.03, -0.94) and the interaction between roommate baseline BMI and time was significant (ß = 0.08, 95% CI = 0.03, 0.13; S4 Table). This suggests that participants assigned to a roommate with a higher BMI had a lower initial BMI, but gained more weight between T1 and T4.

When participant weight was predicted by their roommate weight, the interaction between roommate weight and time was significant (ß = 0.04, 95% CI = 0.01, 0.07; S5 Table). This model suggests that a participant with a baseline weight of 65 would be expected to lose 0.1 kg between T2 and T4 if assigned to a roommate with a weight of 50 kg, and gain 1.1 kg between T2 and T4 if assigned to a roommate with a weight of 80 kg.

When the models were stratified by sex, roommate baseline BMI was significantly associated with the BMI trajectory of females (n = 84, ß = 0.06, 95% CI = 0.01, 0.10; S6 Table) but not males (n = 20, ß = 0.05, 95% CI = -0.06, 0.17; S7 Table). While this effect was not “significant” for males, the beta estimate of 0.05 was consistent with that found in the other models.

When examining the Fall and Spring semesters separately, the estimated effects of roommate BMI on participant BMI were a similar magnitude to the effect for the full-year analyses (Fall: n = 93, ß = 0.04, 95% CI = 0.00, 0.08, S8 Table; Spring: n = 68, ß = 0.06, 95% CI = 0.02, 0.10, S9 Table). The effect was significant for the Spring, but not for the Fall.

The final model evaluates the possibility that shared environment could have been responsible for the above findings indicative of roommate influence. When the 104 floormate pairs were examined, no association was found between participant BMI trajectory and participant baseline BMI (β = 0.01, 95% CI = 0.00, 0.02) or their floormate baseline BMI (β = -0.01, 95% CI = -0.04, 0.03; S10 Table). The near-zero, non-significant estimate for floormate BMI suggests that being on the same floor was not sufficient to produce the pattern of peer influence we observe.

## Discussion

This study examined roommate influence on body mass index (BMI) change using a natural experiment of randomly assigned roommates combined with an innovative floormate analysis to rule out selection or shared environment as competing explanations. The findings suggest that first-year college students’ weight trajectories are influenced by the weight status of their randomly assigned roommate. For every 5 kg/m^2^ greater baseline BMI of a roommate at the start of the Fall 2015 semester, a participant would be expected to gain an additional 0.3 kg/m^2^ between the end of the Fall 2015 and the end of the Spring 2016 semesters. While the magnitude of the effect is modest, considered at the population level, these results could have significant impacts on weight patterning. These findings also suggest that the similarity observed among friends cannot be attributed solely to selection of friends based on BMI or other unmeasured variables. Controlling for clustering by campus, combined with null results in the floormate analysis, helps to rule out shared environment as an alternative explanation. Finally, as pre-existing friendships were excluded from the study, this study indicates that peer influence on BMI may occur via *proximity* mechanisms, rather than longer term *friendship* selection mechanisms.

In light of these findings, it is worth reflecting more deeply on the nature of peer influence within roommate relationships in particular. Although we find evidence of peer influence among roommates, the mechanisms by which roommates influence each other’s weight trajectories are not yet well understood. One prior study finds that the extent of shared activities among first-year college roommates decreases throughout the first academic year [73], which may suggest that less time is spent with roommates after more ‘desirable’ friendships are formed later in the semester. While roommates may not be first-year college students’ most desirable friends, they are required to live together. Compared to friends, roommates may be more likely to observe each other’s morning and evening routines. Peer influence via mechanisms such as sleep routines, night eating, screen time, and breakfast eating may be more relevant for roommates than friends. Students may also spend time with their roommate when doing less social activities (e.g., studying, sleeping), and spend time with friends for social activities (e.g., eating, socializing). As such, friends may see “frontstage” activities (such as alcohol intake at a party) while roommates may see the more typical “backstage” activities (such as daily alcohol intake). The mechanisms for BMI influence among roommates and friends may be different given these expected differences in shared activities among roommates and friends. Notably, even within friendships, which are far more often the focus of inquiry, the mechanisms by which friends influence each other’s weight trajectories are still unclear. Thus, additional research on the mechanisms behind peer influence among both roommates and friends is warranted, particularly with an eye toward differences in relational properties that shape which mechanisms are activated.

While this study examined students, roommates are also prevalent outside of the college context. The prevalence of a roommate living arrangement is greatest among 18–24 year-olds with some college education who are continuing their education, however roommates among adults outside this age range are also common [74, 75], including among retirees [76]. While we would anticipate peer influence among other (non-college) roommates, the lack of random assignment makes parsing out selection and influence effects more difficult for roommates in other environments. Thus, other research designs may be needed to understand influence among non-randomly assigned roommates.

Weight discrimination may also partially explain our findings. Students who experience weight discrimination may gain more weight than their counterparts for many reasons [77]. For instance, weight discrimination is associated with unhealthy weight control behaviors [78], higher caloric consumption [79], and less exercise [79]. It may be that when a participant's roommate experiences weight discrimination, the roommate *and* the participant engage in unhealthy weight-related behaviors. Minimizing weight discrimination and encouraging self-acceptance may be effective at minimizing weight gain for those at higher BMIs, *and* their roommates.

Regarding females, our findings align with Yakusheva et al. (2014), who also reported an association between participant and roommate weight among females. Our sample size for males was too small to be certain that the non-significant association for male BMI trajectory was due to a true null effect (as reported by Yakusheva et al. (2014) [59]) or not. One study which examined friendship networks (rather than roommates) found null results when testing if sex affects susceptibility to peer influence on BMI [3]. Given sex differences in weight-related behaviors, it is plausible that peer influence is indeed sex-specific in some circumstances. For instance, feeling or experiencing weight discrimination is more prevalent among females than males [80] and female college students are more likely to overestimate their BMI and try to lose weight than males [81]. The amount of weight gained over college may also differ by sex. Some studies suggest that males have a greater increase in BMI than females during the four years of college [82, 83], however other studies suggest that the extent of weight gain over the first year of college is similar among males and females [84–87]. As such it remains unclear if the effect of roommate influence on BMI is sex-specific as suggested by Yakusheva, or not. Additional research is needed in this regard, and with particular attention to relationship type (e.g., friends, roommates, and other types of relationships).

Our study documented influence among first-year university students. The overall findings from this study are consistent with a prior roommate study [59] and other social network studies related to weight [3, 6]. Given accumulating evidence that roommates influence each other's weight trajectories, interventions encouraging healthful lifestyles at the roommate level may be worth considering. It would be useful to determine how roommates influence each other's BMI trajectories over longer periods of time. In particular, universities which provide on-campus housing to students until graduation could follow randomized roommate pairs to determine if the influence effect observed during the first year of university remains until graduation. This study shows that those in close proximity, who are not necessarily friends, influence each other’s BMI change. This study showed no BMI influence among less proximate individuals (floormates), indicating a certain ‘closeness’ is necessary for influence to occur. Increased knowledge of the mechanisms by which proximate individuals influence each other would not only be helpful for preventing obesity, but could potentially be applied to develop interventions encouraging healthful behaviors for other populations who spend time in close proximity (e.g., office co-workers, active members of faith-based communities).

### Limitations

Selection bias may be a limitation given that participants knew that their anthropometrics would be obtained at each survey. The participants who stayed in the study may have had different BMI trajectories than those who were lost to follow-up. The sample size (n = 104) was limited; many participants were excluded due to missing a Spring survey, anthropometrics, or roommate information. Participants whose roommate also completed the baseline survey and anthropometrics may not be representative of other roommates. When comparing the participants included in the main analysis of this study to those who completed baseline assessments, there were no differences in race/ethnicity, financial aid status, or BMI. However, a higher proportion of females and participants from one campus were included in this study than completed baseline assessments.

This study assumed that administrative assignment of roommates effectively created an experimental design with random assignment, thereby eliminating confounds arising through selection. There was some suggestion that not all of the roommates may have been randomly assigned; roommates’ race/ethnicity was significantly more similar than floormates’ race/ethnicity (P = 0.048). The university did not ask about students’ weight when assigning roommates, however it is possible that some survey questions could have been associated with BMI (e.g., sleep times). Students were also able to connect with future roommates before starting university, which may have introduced non-random elements. While roommates were randomly assigned within residence halls, the residence halls themselves were grouped by college. Thus, only students with majors in the same academic college were eligible to be roommates, thereby limiting the extent of possible randomization for roommates and confounds majors with campus. Still, this would only confound our inferences if major were associated with the strength or activation of peer influence on BMI, which is not something we would expect. In terms of shared environment, some of the students in the floormate analysis did not reside on the same floor. These students’ environment may have been more different than students who resided on the same floor. Finally, the findings from this study are from a sample of one cohort of first-year students at a single university and may not be generalizable to others.

## Conclusion

We found roommates significantly influenced each other’s weight change over time. These findings provide evidence that one’s associates have an independent and potentially important influence on weight change.

## Supporting information

S1 Table The association of participant BMI change at a large southwestern university over the 2015–2016 academic year and roommate baseline BMI when time interactions for campus and sex included in model (model B; n = 104). (DOCX)

S2 Table The association of participant BMI change at a large southwestern university over the 2015–2016 academic year and roommate baseline BMI when controlled for residence hall (model C; n = 104). (DOCX)

S3 Table The association of participant BMI change at a large southwestern university over the 2015–2016 academic year and roommate baseline weight status (model D; n = 104). (DOCX)

S4 Table The association of participant BMI change at a large southwestern university over the 2015–2016 academic year when participant baseline BMI included as a response variable, rather than a predictor (model E; n = 373). (DOCX)

S5 Table The association of participant weight change at a large southwestern university over the 2015–2016 academic year and roommate baseline BMI (model F; n = 104). (DOCX)

S6 Table The association of female participants BMI change at a large southwestern university over the 2015–2016 academic year and roommate baseline BMI (model G; Female; n = 84). (DOCX)

S7 Table The association of male participants BMI change at a large southwestern university over the 2015–2016 academic year and roommate baseline BMI (model G; male; n = 20). (DOCX)

S8 Table The association of participant BMI change at a large southwestern university over the Fall semester and roommate BMI at the start of Fall (model H; n = 93). (DOCX)

S9 Table The association of participant BMI change at a large southwestern university over the Spring semester and roommate BMI at the start of Spring (model I; n = 68). (DOCX)

S10 Table The association of participant BMI change at a large southwestern university over the 2015–2016 academic year and the baseline BMI of a same-sex participant living in the same residence hall (model J; n = 208). (DOCX)

S1 Data (CSV)
