# Supplementary material for: First-year college students’ weight change is influenced by their randomly assigned roommates’ BMI
Source: PLoS One. 2020 Nov 24;15(11):e0242681. doi: 10.1371/journal.pone.0242681 (PMC7685435; doi:10.1371/journal.pone.0242681)
Supplement: S1 Table — (DOCX) [file pone.0242681.s001.docx]

**S1 Table.** The association of participant BMI change at a large southwestern university over the 2015-2016 academic year and roommate baseline BMI when time interactions for campus and sex included in model (model B; n=104).

|  |  | β | SE | 95% CI | p-value |
| --- | --- | --- | --- | --- | --- |
| Intercept |  | 25.53 | 0.19 | (25.17, 25.89) | **<0.001** |
| Linear time trend^A^ |  | 0.34 | 0.11 | (0.13, 0.56) | **0.003** |
| Sex | Female | (ref) |  |  |  |
|  | Male | -0.43 | 0.30 | (-1.01, 0.16) | 0.157 |
| Race/ethnicity | Non-Hispanic White | (ref) |  |  |  |
|  | Other | -0.29 | 0.20 | (-0.67, 0.10) | 0.149 |
| Pell grant recipient | No | (ref) |  |  |  |
|  | Yes | 0.06 | 0.19 | (-0.32, 0.44) | 0.770 |
| Campus | A | (ref) |  |  |  |
|  | B | 0.12 | 0.26 | (-0.38, 0.62) | 0.638 |
| Participant BMI @Time 1 |  | 0.98 | 0.02 | (0.93, 1.02) | **<0.001** |
| Roommate BMI @Time 1 |  | 0.03 | 0.02 | (-0.02, 0.08) | 0.223 |
| Time^A^: Campus = B |  | -0.26 | 0.21 | (-0.66, 0.15) | 0.224 |
| Time^A^: Sex = Male |  | -0.19 | 0.23 | (-0.63, 0.25) | 0.401 |
| Time^A^ : Participant BMI @Time 1 |  | 0.02 | 0.02 | (-0.02, 0.06) | 0.293 |
| Time^A^ : Roommate BMI @Time 1 |  | 0.06 | 0.02 | (0.02, 0.10) | **0.005** |

^A^ The time variable in the model is from Time 2 (0, end of Fall semester) to Time 4 (1, end of Spring semester)
Boldface indicates statistical significance (p<0.05)
