# Supplementary material for: First-year college students’ weight change is influenced by their randomly assigned roommates’ BMI
Source: PLoS One. 2020 Nov 24;15(11):e0242681. doi: 10.1371/journal.pone.0242681 (PMC7685435; doi:10.1371/journal.pone.0242681)
Supplement: S2 Table — (DOCX) [file pone.0242681.s002.docx]

**S2 Table.** The association of participant BMI change at a large southwestern university over the 2015-2016 academic year and roommate baseline BMI when controlled for residence hall (model C; n=104).

|  |  | β | SE | 95% CI | p-value |
| --- | --- | --- | --- | --- | --- |
| Intercept |  | 25.67 | 0.19 | (25.31, 26.03) | **<0.001** |
| Linear time trend^A^ |  | 0.25 | 0.09 | (0.07, 0.43) | **0.008** |
| Sex | Female | (ref) |  |  |  |
|  | Male | -0.48 | 0.26 | (-1.00, 0.04) | 0.075 |
| Race/ethnicity | Non-Hispanic White | (ref) |  |  |  |
|  | Other | -0.33 | 0.19 | (-0.70, 0.05) | 0.094 |
| Pell grant recipient | No | (ref) |  |  |  |
|  | Yes | 0.05 | 0.19 | (-0.33, 0.43) | 0.794 |
| Residence Hall ^B^ | A | (ref) |  |  |  |
|  | B | -0.42 | 0.28 | (-0.97, 0.13) | 0.142 |
|  | C | 0.28 | 0.34 | (-0.39, 0.95) | 0.420 |
|  | D | 0.05 | 0.43 | (-0.79, 0.90) | 0.900 |
|  | E | -1.02 | 0.43 | (-1.86, -0.17) | **0.023** |
|  | F | -0.11 | 0.55 | (-1.18, 0.95) | 0.835 |
|  | G | 0.57 | 0.75 | (-0.91, 2.04) | 0.456 |
| Participant BMI @Time 1 |  | 0.97 | 0.02 | (0.93, 1.02) | **<0.001** |
| Roommate BMI @Time 1 |  | 0.03 | 0.02 | (-0.02, 0.07) | 0.284 |
| Time^A^ : Participant BMI @ Time 1 |  | 0.02 | 0.02 | (-0.02, 0.06) | 0.370 |
| Time^A^ : Roommate BMI @Time 1 |  | 0.06 | 0.02 | (0.02, 0.10) | **0.007** |

^A^ The time variable in the model is from Time 2 (0, end of Fall semester) to Time 4 (1, end of Spring semester)
^B^ Given low counts within some residence halls, the statistical significant result from residence hall D cannot be meaningfully interpreted. Residence halls A to G contributed 60, 10, 2, 6, 4, 6, and 16 participants respectively.
Boldface indicates statistical significance (p<0.05)
