# Supplementary material for: First-year college students’ weight change is influenced by their randomly assigned roommates’ BMI
Source: PLoS One. 2020 Nov 24;15(11):e0242681. doi: 10.1371/journal.pone.0242681 (PMC7685435; doi:10.1371/journal.pone.0242681)
Supplement: S3 Table — (DOCX) [file pone.0242681.s003.docx]

**S3 Table.** The association of participant BMI change at a large southwestern university over the 2015-2016 academic year and roommate baseline weight status (model D; n=104).

|  |  | β | SE | 95% CI | p-value |
| --- | --- | --- | --- | --- | --- |
| Intercept |  | 25.41 | 0.19 | (25.04, 25.78) | **<0.001** |
| Linear time trend^A^ |  | 0.03 | 0.11 | (-0.18, 0.24) | 0.789 |
| Sex | Female | (ref) |  |  |  |
|  | Male | -0.44 | 0.27 | (-0.98, 0.09) | 0.110 |
| Race/ethnicity | Non-Hispanic White | (ref) |  |  |  |
|  | Other | -0.27 | 0.19 | (-0.66, 0.11) | 0.162 |
| Pell grant recipient | No | (ref) |  |  |  |
|  | Yes | 0.07 | 0.19 | (-0.31, 0.44) | 0.727 |
| Campus | A | (ref) |  |  |  |
|  | B | 0.00 | 0.24 | (-0.46, 0.47) | 0.989 |
| Participant BMI @ Time 1 |  | 0.98 | 0.02 | (0.93, 1.02) | **<0.001** |
| Roommate WS^B^ @ Time 1 |  | 0.31 | 0.21 | (-0.11, 0.73) | 0.153 |
| Time^A^ : Participant BMI @ Time 1 |  | 0.02 | 0.02 | (-0.02, 0.06) | 0.317 |
| Time^A^ : Roommate WS^B^ @ Time 1 |  | 0.49 | 0.19 | (0.12, 0.85) | **0.011** |

^A^ The time variable in the model is from Time 2 (0, end of Fall semester) to Time 4 (1, end of Spring semester)
^B^ WS= Weight Status (overweight/obese or not)
Boldface indicates statistical significance (p<0.05)
