# Supplementary material for: First-year college students’ weight change is influenced by their randomly assigned roommates’ BMI
Source: PLoS One. 2020 Nov 24;15(11):e0242681. doi: 10.1371/journal.pone.0242681 (PMC7685435; doi:10.1371/journal.pone.0242681)
Supplement: S4 Table — (DOCX) [file pone.0242681.s004.docx]

**S4 Table.** The association of participant BMI change at a large southwestern university over the 2015-2016 academic year when participant baseline BMI included as a response variable, rather than a predictor (model E; n=373).

|  |  | β | SE | 95% CI | p-value |
| --- | --- | --- | --- | --- | --- |
| Intercept |  | 22.02 | 1.06 | (19.95, 24.08) | **<0.001** |
| Linear time trend^A^ |  | 0.66 | 0.11 | (0.44, 0.88) | **<0.001** |
| Sex | Female | (ref) |  |  |  |
|  | Male | 3.88 | 2.12 | (-0.35, 8.10) | 0.074 |
| Race/ethnicity | Non-Hispanic White | (ref) |  |  |  |
|  | Other | -0.01 | 0.18 | (-0.38, 0.36) | 0.959 |
| Pell grant recipient | No | (ref) |  |  |  |
|  | Yes | 0.02 | 0.17 | (-0.31, 0.35) | 0.908 |
| Campus | A | (ref) |  |  |  |
|  | B | -0.86 | 1.89 | (-4.61, 2.89) | 0.650 |
| Roommate BMI @ Time 1 |  | -0.98 | 0.02 | (-1.03, -0.94) | **<0.001** |
| Time^A^ : Roommate BMI @ Time 1 |  | 0.08 | 0.03 | (0.03, 0.13) | **0.001** |

^A^ The time variable in the model is from Time 1 (0, start of Fall semester) to Time 4 (1, end of Spring semester)
Boldface indicates statistical significance (p<0.05)
