# Supplementary material for: First-year college students’ weight change is influenced by their randomly assigned roommates’ BMI
Source: PLoS One. 2020 Nov 24;15(11):e0242681. doi: 10.1371/journal.pone.0242681 (PMC7685435; doi:10.1371/journal.pone.0242681)
Supplement: S5 Table — (DOCX) [file pone.0242681.s005.docx]

**S5 Table.** The association of participant weight change at a large southwestern university over the 2015-2016 academic year and roommate baseline BMI (model F; n=104).

|  |  | β | SE | 95% CI | *P* |
| --- | --- | --- | --- | --- | --- |
| Intercept |  | 2.37 | 2.24 | (-1.96, 6.71) | 0.290 |
| Linear time trend^A^ |  | -4.13 | 1.50 | (-7.05, -1.21) | **0.007** |
| Sex | Female |  |  |  |  |
|  | Male | -0.81 | 1.00 | (-2.78, 1.15) | 0.418 |
| Race/ethnicity | Non-Hispanic White |  |  |  |  |
|  | Other | -0.68 | 0.56 | (-1.78, 0.42) | 0.228 |
| Pell Grant recipient | No |  |  |  |  |
|  | Yes | 0.00 | 0.55 | (-1.09, 1.08) | 0.993 |
| Campus | A |  |  |  |  |
|  | B | 0.30 | 0.67 | (-1.02, 1.62) | 0.654 |
| Participant weight @ Time 1 |  | 0.98 | 0.02 | (0.93, 1.02) | **<0.001** |
| Roommate weight @ Time 1 |  | 0.01 | 0.02 | (-0.03, 0.05) | 0.659 |
| Time^A^ : Participant weight @Time 1 |  | 0.03 | 0.02 | (0.00, 0.07) | 0.068 |
| Time^A^ : Roommate weight @Time 1 |  | 0.04 | 0.02 | (0.01, 0.07) | **0.027** |

^A^ The time variable in the model is from Time 2 (0, end of Fall semester) to Time 4 (1, end of Spring semester)
Boldface indicates statistical significance (p<0.05)
