# Supplementary material for: First-year college students’ weight change is influenced by their randomly assigned roommates’ BMI
Source: PLoS One. 2020 Nov 24;15(11):e0242681. doi: 10.1371/journal.pone.0242681 (PMC7685435; doi:10.1371/journal.pone.0242681)
Supplement: S6 Table — (DOCX) [file pone.0242681.s006.docx]

**S6 Table.** The association of female participants BMI change at a large southwestern university over the 2015-2016 academic year and roommate baseline BMI (model G; Female; n=84).

|  |  | β | SE | 95% CI | *P* |
| --- | --- | --- | --- | --- | --- |
| Intercept |  | 25.62 | 0.14 | (25.34, 25.90) | **<0.001** |
| Linear time trend^A^ |  | 0.30 | 0.10 | (0.12, 0.49) | **0.002** |
| Race/ethnicity | Non-Hispanic White | (ref) |  |  |  |
|  | Other | -0.14 | 0.17 | (-0.48, 0.20) | 0.425 |
| Pell Grant recipient | No | (ref) |  |  |  |
|  | Yes | -0.09 | 0.18 | (-0.43, 0.26) | 0.628 |
| Campus | A | (ref) |  |  |  |
|  | B | 0.00 | 0.19 | (-0.37, 0.37) | 0.997 |
| Participant BMI @ Time 1 |  | 1.03 | 0.02 | (0.98, 1.07) | **<0.001** |
| Roommate BMI @ Time 1 |  | 0.03 | 0.02 | (-0.01, 0.08) | 0.130 |
| Time^A^ : Participant BMI @ Time 1 |  | 0.04 | 0.02 | (0.00, 0.08) | **0.045** |
| Time^A^ : Roommate BMI @ Time 1 |  | 0.06 | 0.02 | (0.01, 0.10) | **0.010** |

^A^ The time variable in the model is from Time 2 (0, end of Fall semester) to Time 4 (1, end of Spring semester)
Boldface indicates statistical significance (p<0.05)
