# Supplementary material for: First-year college students’ weight change is influenced by their randomly assigned roommates’ BMI
Source: PLoS One. 2020 Nov 24;15(11):e0242681. doi: 10.1371/journal.pone.0242681 (PMC7685435; doi:10.1371/journal.pone.0242681)
Supplement: S7 Table — (DOCX) [file pone.0242681.s007.docx]

**S7 Table.** The association of male participants BMI change at a large southwestern university over the 2015-2016 academic year and roommate baseline BMI (model G; male; n=20).

|  |  | β | SE | 95% CI | *P* |
| --- | --- | --- | --- | --- | --- |
| Intercept |  | 25.16 | 0.44 | (24.34, 25.98) | **<0.001** |
| Linear time trend^A^ |  | 0.11 | 0.25 | (-0.35, 0.58) | 0.652 |
| Race/ethnicity | Non-Hispanic White | (ref) |  |  |  |
|  | Other | -0.23 | 0.34 | (-0.99, 0.53) | 0.531 |
| Pell Grant recipient | No | (ref) |  |  |  |
|  | Yes | 0.15 | 0.28 | (-0.47, 0.77) | 0.609 |
| Campus | A | (ref) |  |  |  |
|  | B | 0.21 | 0.71 | (-1.27, 1.69) | 0.771 |
| Participant BMI @ Time 1 |  | 0.74 | 0.07 | (0.58, 0.90) | **<0.001** |
| Roommate BMI @ Time 1 |  | -0.08 | 0.07 | (-0.24, 0.08) | 0.322 |
| Time^A^ : Participant BMI @ Time 1 |  | -0.10 | 0.07 | (-0.23, 0.03) | 0.163 |
| Time^A^ : Roommate BMI @ Time 1 |  | 0.05 | 0.06 | (-0.06, 0.17) | 0.390 |

^A^ The time variable in the model is from Time 2 (0, end of Fall semester) to Time 4 (1, end of Spring semester)
Boldface indicates statistical significance (p<0.05)
