# Supplementary material for: First-year college students’ weight change is influenced by their randomly assigned roommates’ BMI
Source: PLoS One. 2020 Nov 24;15(11):e0242681. doi: 10.1371/journal.pone.0242681 (PMC7685435; doi:10.1371/journal.pone.0242681)
Supplement: S8 Table — (DOCX) [file pone.0242681.s008.docx]

**S8 Table.** The association of participant BMI change at a large southwestern university over the Fall semester and roommate BMI at the start of Fall (model H; n=93).

|  |  | β | SE | 95% CI | *P* |
| --- | --- | --- | --- | --- | --- |
| Intercept |  | 25.53 | 0.14 | (25.25, 25.82) | **<0.001** |
| Sex | Female | (ref) |  |  |  |
|  | Male | -0.31 | 0.21 | (-0.73, 0.12) | 0.156 |
| Race/ethnicity | Non-Hispanic White | (ref) |  |  |  |
|  | Other | -0.12 | 0.17 | (-0.46, 0.22) | 0.484 |
| Pell Grant recipient | No | (ref) |  |  |  |
|  | Yes | -0.17 | 0.18 | (-0.52, 0.18) | 0.344 |
| Campus | A | (ref) |  |  |  |
|  | B | 0.22 | 0.19 | (-0.16, 0.59) | 0.261 |
| Participant BMI @ Time 1 |  | 1.00 | 0.02 | (0.96, 1.04) | **<0.001** |
| Roommate BMI @ Time 1 |  | 0.04 | 0.02 | (0.00, 0.08) | 0.061 |

Boldface indicates statistical significance (p<0.05)
