# Supplementary material for: First-year college students’ weight change is influenced by their randomly assigned roommates’ BMI
Source: PLoS One. 2020 Nov 24;15(11):e0242681. doi: 10.1371/journal.pone.0242681 (PMC7685435; doi:10.1371/journal.pone.0242681)
Supplement: S9 Table — (DOCX) [file pone.0242681.s009.docx]

**S9 Table.** The association of participant BMI change at a large southwestern university over the Spring semester and roommate BMI at the start of Spring (model I; n=68).

|  |  | β | SE | 95% CI | *P* |
| --- | --- | --- | --- | --- | --- |
| Intercept |  | 25.37 | 0.14 | (25.09, 25.65) | **0.007** |
| Sex | Female | (ref) |  |  |  |
|  | Male | -0.59 | 0.22 | (-1.02, -0.16) | **0.008** |
| Race/ethnicity | Non-Hispanic White | (ref) |  |  |  |
|  | Other | 0.03 | 0.17 | (-0.31, 0.36) | 0.879 |
| Pell Grant recipient | No | (ref) |  |  |  |
|  | Yes | -0.17 | 0.17 | (-0.51, 0.16) | 0.308 |
| Campus | A | (ref) |  |  |  |
|  | B | -0.22 | 0.20 | (-0.61, 0.17) | 0.255 |
| Participant BMI @ Time 3 |  | 1.02 | 0.02 | (0.99, 1.06) | **<0.001** |
| Roommate BMI @ Time 3 |  | 0.06 | 0.02 | (0.02, 0.10) | **0.002** |

Boldface indicates statistical significance (p<0.05)
