# Supplementary material for: First-year college students’ weight change is influenced by their randomly assigned roommates’ BMI
Source: PLoS One. 2020 Nov 24;15(11):e0242681. doi: 10.1371/journal.pone.0242681 (PMC7685435; doi:10.1371/journal.pone.0242681)
Supplement: S10 Table — (DOCX) [file pone.0242681.s010.docx]

**S10 Table.** The association of participant BMI change at a large southwestern university over the 2015-2016 academic year and the baseline BMI of a same-sex participant living in the same residence hall (model J; n=208).

|  |  | β | SE | 95% CI | p-value |
| --- | --- | --- | --- | --- | --- |
| Intercept |  | 25.48 | 0.03 | (25.42, 25.55) | **<0.001** |
| Linear time trend^A^ |  | 0.21 | 0.04 | (0.13, 0.30) | **<0.001** |
| Sex | Female | (ref) |  |  |  |
|  | Male | -0.17 | 0.04 | (-0.26, -0.09) | **<0.001** |
| Race/ethnicity | Non-Hispanic White | (ref) |  |  |  |
|  | Other | -0.14 | 0.03 | (-0.20, -0.07) | **<0.001** |
| Pell grant recipient | No | (ref) |  |  |  |
|  | Yes | 0.01 | 0.04 | (-0.07, 0.08) | 0.844 |
| Campus | A | (ref) |  |  |  |
|  | B | 0.03 | 0.05 | (-0.07, 0.13) | 0.522 |
| Participant BMI @ Time 1 |  | 0.99 | 0.00 | (0.98, 1.00) | **<0.001** |
| Floormate BMI @ Time 1 |  | 0.01 | 0.01 | (-0.01, 0.04) | 0.337 |
| Time^A^ : Participant BMI @ Time 1 |  | 0.01 | 0.01 | (0.00, 0.02) | 0.193 |
| Time^A^ : Floormate BMI @ Time 1 |  | -0.01 | 0.02 | (-0.04, 0.03) | 0.755 |

^A^ The time variable in the model is from Time 2 (0, end of Fall semester) to Time 4 (1, end of Spring semester)
Boldface indicates statistical significance (p<0.05)
